# Trigeminal Nerve Root Demyelination Not Seen in Six Horses Diagnosed with Trigeminal-Mediated Headshaking

**DOI:** 10.3389/fvets.2017.00072

**Published:** 2017-05-15

**Authors:** Veronica L. Roberts, Debra Fews, Jennifer M. McNamara, Seth Love

**Affiliations:** ^1^School of Veterinary Clinical Sciences, University of Bristol, Bristol, Somerset, UK; ^2^Centre for Comparative and Clinical Anatomy, University of Bristol, Bristol, UK; ^3^Department of Neuropathology, University of Bristol Institute of Clinical Neurosciences, Southmead Hospital, Bristol, UK

**Keywords:** trigeminal-mediated, headshaking, idiopathic, horse, nerve root, demyelination

## Abstract

Trigeminal-mediated headshaking is an idiopathic neuropathic facial pain syndrome in horses. There are clinical similarities to trigeminal neuralgia, a neuropathic facial pain syndrome in man, which is usually caused by demyelination of trigeminal sensory fibers within either the nerve root or, less commonly, the brainstem. Our hypothesis was that the neuropathological substrate of headshaking in horses is similar to that of trigeminal neuralgia in man. Trigeminal nerves, nerve roots, ganglia, infraorbital, and caudal nasal nerves from horse abattoir specimens and from horses euthanized due to trigeminal-mediated headshaking were removed, fixed, and processed for histological assessment by a veterinary pathologist and a neuropathologist with particular experience of trigeminal neuralgia histology. No histological differences were detected between samples from horses with headshaking and those from normal horses. These results suggest that trigeminal-mediated headshaking may have a different pathological substrate from trigeminal neuralgia in man.

## Introduction

Headshaking in horses is a syndrome of spontaneous and repetitive movements of the head and neck (1) predominantly in a vertical orientation (2). These movements are often accompanied by signs of nasal irritation (1, 2). The condition is usually worse at exercise (3) but may be seen at rest, in the stable and/or field (2). In the majority (98%) of cases, no physical cause is identified even after extensive clinical investigation (3). It is now widely accepted that most cases of headshaking are due to an idiopathic trigeminal neuropathy (1, 2, 4–9) and the term “trigeminal-mediated headshaking” is the accepted term to describe this condition (10).

The trigeminal nerve in trigeminal-mediated headshakers was shown to be abnormally sensitized, with an abnormally low threshold for activation when somatosensory-evoked potentials were tested (8, 11, 12). In a horse affected seasonally, the activation threshold was normal when the horse was tested out of season and therefore not showing clinical signs (11). This suggests a reversible disease process, although unfortunately the same horse was not tested when showing clinical signs. There is evidence that trigeminal-mediated headshakers suffer facial pain, as signs can be temporarily abolished by infiltration of local anesthetic around the caudal portion of the infraorbital nerve bilaterally (9). They do not respond to analgesia with non-steroidal anti-inflammatory drugs (6) but in some cases respond to carbamazepine and/or cyproheptadine (1, 6) or to neuromodulation by percutaneous electrical nerve stimulation (13), which would be consistent with neuropathic pain.

Trigeminal neuralgia is a human neuropathic facial pain syndrome. It is usually caused by demyelination of trigeminal sensory fibers within either the nerve root or, less commonly, the brainstem (14). In most cases, the trigeminal nerve root demyelination involves the proximal, CNS part of the root and results from compression by an overlying artery or vein. Other causes of trigeminal neuralgia in which demyelination is involved or implicated include multiple sclerosis and, probably, compressive space-occupying masses in the posterior fossa (14). Determination of the underlying pathology has facilitated the development of targeted treatment. Initial histopathological examination of the infraorbital nerve of horses’ euthanized due to trigeminal-mediated headshaking did not reveal any abnormality but a comprehensive examination of the nerve, including the nerve root, was not reported (11).

In this study, we investigated the possibility that the neuropathological substrate of headshaking in horses is similar to that of trigeminal neuralgia in man.

## Materials and Methods

### Histopathological Anatomy in Normal Horses

Cadaver horse heads (four) were collected from an abattoir from horses presented for slaughter. Clinical history was unknown, but all had been assessed as fit for slaughter for human consumption on antemortem inspection. Controls were not age-matched to clinical cases. Heads were removed as routine during carcass preparation and immediately put into ice baths for transport. Dissection was performed within 3 h of slaughter.

**Figure 1:**
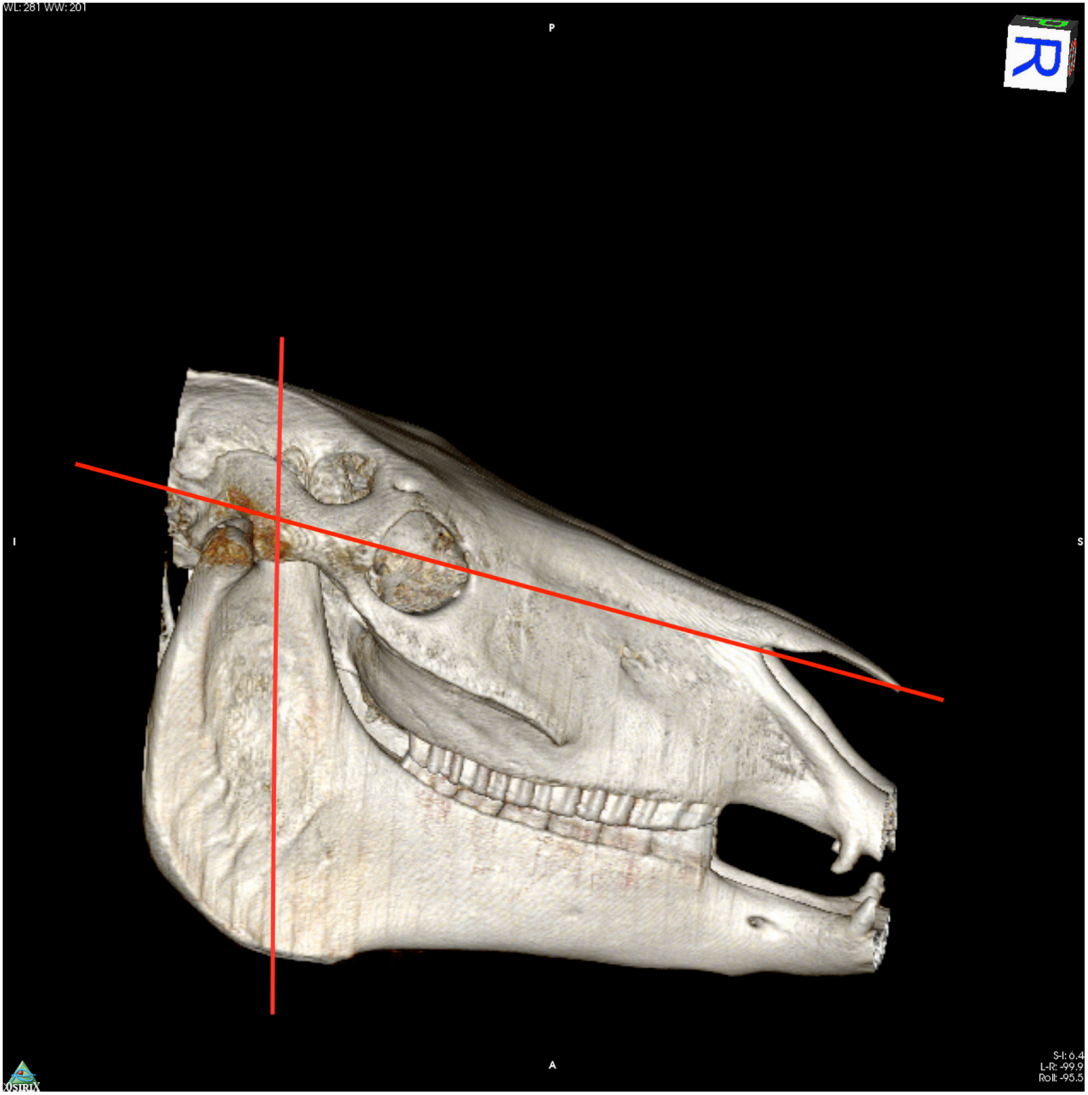
**Reconstructed computed tomography image to illustrate cuts made in specimen dissection**.

To access the caudal portion of the infraorbital nerve, the horses’ heads were cut using a band saw to access the caudal portion of the infraorbital canal. The first cut was a transverse slice that approximately bisected the angle of the mandible and masseteric fossa of each mandible. The second slice was along the length of the head following the line of the facial crest and allowing removal of the nasal and frontal bones (Figure 1). The dorsal roof of the infraorbital canal was removed. The caudal third of the infraorbital nerve was transected rostral to the point parallel to tooth 110/210, back to the site of emergence from the brain. The trigeminal nerve on both sides of the head was removed to include the trigeminal nerve root, ganglion, infraorbital, and caudal nasal nerves. Specimens were fixed in 10% neutral buffered formalin, routinely processed to paraffin wax, sectioned at 4 μm, then stained with hematoxylin and eosin, Luxol fast blue/Cresyl violet, solochrome cyanin, or Palmgren silver impregnation.

### Histopathological Anatomy in Trigeminal-Mediated Headshakers

Suitable cases (six) were horses diagnosed with trigeminal-mediated headshaking who had been euthanized following failure to respond to treatment. Owners gave informed consent for the use of their horses’ bodies for this study. Dissection was performed either within 1 h after euthanasia or up to 3 h later, when heads were again stored in ice.

Dissection and slide preparation were performed by the same technique as for the abattoir specimens. As some cases had undergone “coil compression” of the infraorbital nerve for treatment of headshaking (2 and 9), the section of the infraorbital nerve affected by the surgery (level with the rostral border of tooth 111 and 211) was not used in this study.

The histological appearance of samples taken from horses with headshaking was compared to the samples taken from normal horses, by a veterinary pathologist and a neuropathologist with particular experience of trigeminal neuralgia histology. Each sample was sectioned and examined at multiple levels.

## Results

There were four assumed normal abattoir specimens and six clinical cases (20 nerves). Of the latter, three had undergone “coil compression” surgery, which had failed to alleviate clinical signs. The portion of the nerve affected by this procedure was not used in this study for any of the specimens (a 2-cm stretch of the infraorbital nerve, level with the rostral border of tooth 111 and 211).

**Figure 2:**
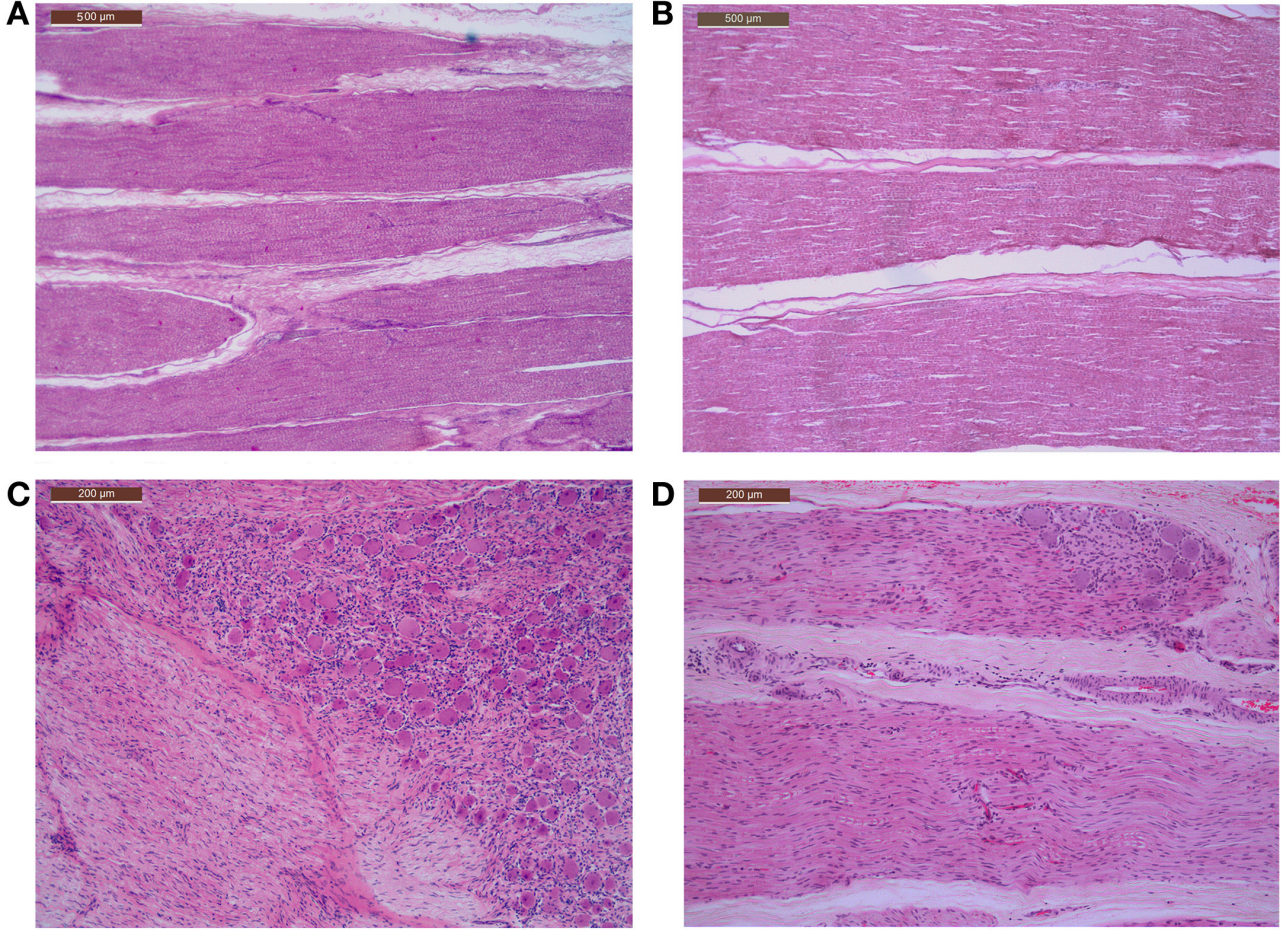
**Microscopic features of the trigeminal nerve roots and caudal nasal nerves, including portions of the pterygopalatine ganglion, of control and affected horses, showing normal axons and myelination within the nerves and normal ganglion cells and architecture within the ganglia**. **(A)** Photomicrograph control horse normal trigeminal nerve root H&E stain. **(B)** Affected horse normal trigeminal nerve root H&E stain. **(C)** Photomicrograph control horse including a portion of normal caudal nasal nerve and pterygopalatine ganglion H&E stained. **(D)** Affected horse including a portion of normal caudal nasal nerve and pterygopalatine ganglion H&E stain.

**Figure 3:**
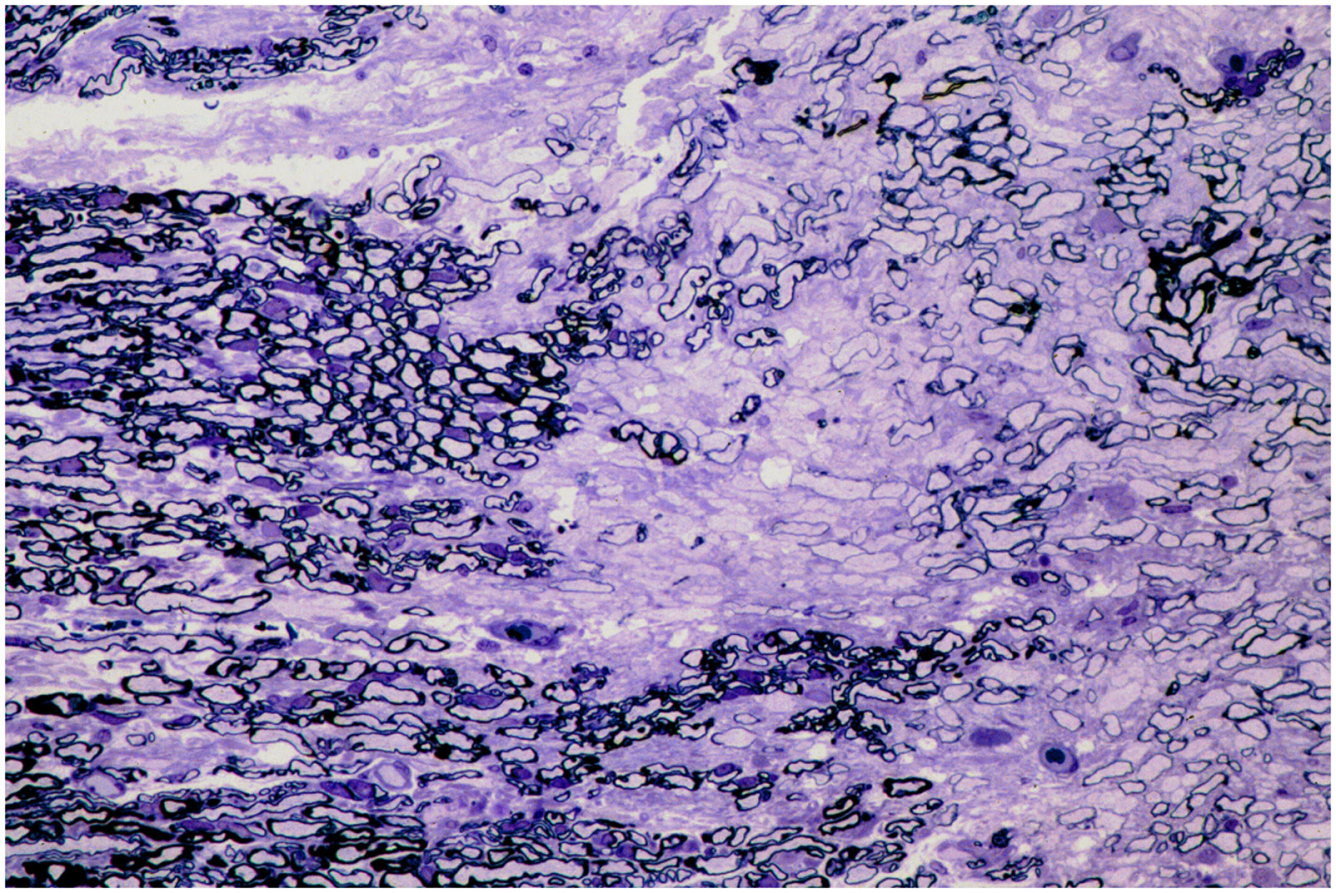
**Photomicrograph of semi-thin sections through a region of trigeminal nerve root compression in a human patient reveals a zone of demyelination within the proximal, CNS part of the nerve root, close to the junction with the PNS**. Several thinly myelinated fibers are present within the zone of demyelination. Toluidine blue. Reproduced with permission from Ref. (14).

No histopathological abnormalities were detected on microscopic examination of the trigeminal nerve root, trigeminal ganglion, infraorbital nerve (excluding segments directly affected by coil placement), and caudal nasal nerve. In particular, there was no increase in cellularity or reduction of myelin staining to suggest demyelination, such as is often observed, usually in the trigeminal nerve root, in people with trigeminal neuralgia (Figures 2 and 3).

## Discussion

These results suggest that, while trigeminal-mediated headshaking in horses has apparent clinical similarity to trigeminal neuralgia in human, it may have a different underlying pathological substrate.

Trigeminal neuralgia in people is a neuropathic facial pain syndrome characterized by a recurrent sudden, severe sharp electric shock-like pain (15). Certainly, trigeminal-mediated headshaking in horses is a facial pain syndrome, with signs of temporary amelioration following infiltration of local anesthetic around the trigeminal nerve (9). Poor response to non-steroidal anti-inflammatory medication but response in some cases to administration of carbamazepine (6) and use of percutaneous electrical nerve stimulation (13) are consistent with the facial pain being neuropathic in these horses.

Both headshaking and trigeminal neuralgia appear to be acquired, although in people usually in an older equivalent population than in horses (15, 16). There are a number of other clinical differences. In trigeminal neuralgia, there is a slight female predominance (14, 15), whereas trigeminal-mediated headshaking may be more common in neutered males (16) although clearly we cannot make a direct comparison here. Trigeminal neuralgia is typically unilateral (14, 15), whereas in horses the trigeminal nerve is affected bilaterally (11), so indeed the pathology could be central. Symptoms of trigeminal neuralgia are usually triggered by light stimuli, such as touching, chewing, talking, and tooth brushing involving specific mucocutaneous areas of the head, oral cavity, or neck: the “trigger zones” (15). Signs of trigeminal-mediated headshaking may be evident at rest but are often elicited by exercise (16), although in some cases signs manifest only when exercise is performed outdoors and not indoors (personal observation). In some cases, signs may occur only during the spring and summer months and may be worsened in bright sunlight and wind (16). This suggests a complex role of the environment in the development of the condition. People may suffer post-herpetic neuralgia (14), but trigeminal-mediated headshaking has been shown to not be associated with latent herpes virus infection (17).

There are also differences at an electrophysiological level. The electrophysiological features of demyelination, such as temporal dispersion, polyphasia, and conduction block, are not seen in affected horses (18). Trigeminal-mediated headshakers instead, demonstrate sensitization of the trigeminal nerve, as evidenced by a low threshold potential for nerve activation when measured using somatosensory-evoked potentials (8, 11, 12). The sensitization appears to be reversible, as it was not evident in a seasonally affected headshaker when tested out of season and free of clinical signs.

Present findings extend those of Aleman et al. (11). There appears to be no structural abnormality in the trigeminal nerve of horses affected by trigeminal-mediated headshaking. A functional, non-structural abnormality may reflect central pathology or membrane instability and raise the possibility that the condition is potentially reversible, as occurs naturally in seasonally affected horses. Limitations of the present study include the small sample size without close age-matching of cases to controls, our assumption that abattoir specimens were not affected by trigeminal-mediated headshaking, and the selection bias for donated affected horses to be non-responders to current available treatments. There was opportunity to blind the pathologists as to origin of specimens and this was not availed of.

Further work should be directed at determining the cause of the functional abnormality and the factors which induce it, as this knowledge would allow the development of targeted treatments.

## Ethics Statement

This study was performed on cadavers of horses’ euthanized for veterinary reasons or for human consumption. Owners of clinical cases gave informed consent for the use of their horses’ bodies in this study. This study was approved by the University of Bristol Ethics Committee.

## Author Contributions

VR is responsible for the conception of this research and acquisition of specimens. All authors contributed equally to project design. VR, DF, and JM performed dissection all on cases together. DF and SL examined the histopathology slides separately and together and prepared the photomicrographs. VR drafted the work, with all authors involved in the final approval of the version to be published. All authors agreed to be accountable for all aspects of the work in ensuring that questions related to the accuracy or integrity of any part of the work are appropriately investigated and resolved.

## Author Note

Part of this research was presented at the meeting of the British Neuropathological Society, 2014.

## Conflict of Interest Statement

The authors declare that the research was conducted in the absence of any commercial or financial relationships that could be construed as a potential conflict of interest.
